# Process optimization, antioxidant, antibacterial, and drug adjuvant properties of bioactive keratin microparticles derived from porcupine (*Hystrix indica*) quills

**DOI:** 10.7717/peerj.15653

**Published:** 2023-08-18

**Authors:** Zahid Majeed, Hoorulain Farhat, Basharat Ahmad, Atia Iqbal, Abu ul Hassan Faiz, Mater H. Mahnashi, Ali O. Alqarni, Omaish Alqahtani, Amer Al Ali, Aiman M. Momenah

**Affiliations:** 1 Department of Biotechnology, Faculty of Science, The University of Azad Jammu and Kashmir, Muzaffarabad, Pakistan; 2 Department of Zoology, Faculty of Science, The University of Azad Jammu and Kashmir, Muzaffarabad, Pakistan; 3 Department of Zoology, The University of Azad Jammu and Kashmir, Muzaffarabad, Pakistan; 4 Department of Microbiology and Molecular Genetics, The Women University, Multan, Pakistan; 5 Department of Zoology, Faculty of Science and Technology, Women University of Azad Jammu and Kashmir, Bagh, Pakistan; 6 Department of Pharmaceutical Chemistry, Najran University, Najran, Saudi Arabia; 7 Department of Pharmacognosy, College of Pharmacy, Najran University, Najran, Saudi Arabia; 8 Department of Clinical Laboratory Sciences, Faculty of Applied Medical Sciences, University of Bisha, Al Nakhil Bisha, Saudi Arabia; 9 Department of Microbiology, Faculty of Medicine, Umm Al-Qura University, Makkah, Saudi Arabia

**Keywords:** Keratin microparticles, Lipids, Antioxidant, Antibacterial, Adjuvant, Porcupine

## Abstract

A structural protein called keratin is often employed in the medical industry to create medication carriers. Process improvement, antioxidant, antibacterial, and adjuvant drug studies of synthetic bioactive keratin microparticles made from lipids and keratin derived from porcupine (*Hystrix indica*) quills are the main objectives of this study. After coating the keratin microparticles with lipids which were obtained from the same porcupine quills, the bioactive keratin microparticles were produced. The response surface technique was applied to optimize the conditions for extraction of the keratin protein and sizing of the keratin microparticles. An infrared spectroscopy was used to analyze the chemical shifts in compositions of keratin microparticles while the optical microscopy was used to measure the size of the keratin microparticles. The results of this work revealed that a yield 27.36 to 42.25% of the keratin protein could be obtained from porcupine quills. The keratin microparticles were sized between 60.65 and 118.87 µm. Through response surface optimization, mercaptoethanol and urea were shown to be the main variables which positively affected the yield and the size of the keratin protein. The lipid stacking on the keratin microparticles’ surface was confirmed by infrared spectroscopy. The 2,2′-azinobis-(3-ethylbenzothiazoline-6-sulphonate) assay confirmed the keratin microparticle’s antioxidant activity of 29.83%. Compared to lipid alone, the antibacterial properties of the keratin microparticles against *Escherichia coli*—a gram-negative—and *Staphylococcus aureus*—a gram-positive—bacteria enhanced by up to 55% following the coating of the microparticles with the lipids. The pharmacological action against these bacterial species was further improved by the lipid-loaded erythromycin that was carried on the surface of keratin microparticles. This work has demonstrated the design and uses of the keratin microparticles obtained from porcupine quills for clinical applications.

## Introduction

In the entire world, antibiotic resistance is a really serious issue. As germs acquire resistance to medications, many of them lose their effectiveness. Between 1960 and 2011, very few novel antibiotic families were successfully developed and marketed. As a result, super germs that are extremely resistant to diseases are currently the subject of extensive research. The particle surface is essential for the delivery of medicines to their targeted targets. Controlling the opsonization process by surface modification is crucial, as it enhances the surface qualities or coating modifications using polymers.

Keratin is a fibrous and resistant structural protein that, after cellulose and chitin, is the third most prevalent polymer in nature. Keratin can act as an effective barrier even against microbial attack because microbial destruction of keratin is rare in nature (Lange, Huang & Busk, 2016). Keratin has a strong structural makeup. It is also extremely stable and insoluble in the majority of organic solvents. Additionally, it is resistant to proteolytic enzymes. Keratin’s high cysteine concentration confers chemical and mechanical resilience.

The sulphide and disulphide linkages that are produced by the covalent bonds between cysteine and cysteine have an impact on the physicochemical properties of keratin (Chilakamarry et al., 2021). Because of its appealing biochemical and structural characteristics, keratin-based materials are highly suited to biomedical, consumer, and defense applications that need material multifunctionality (Dickerson et al., 2013). Keratin has been used in a variety of processes recently, including wound healing, nerve and bone regeneration, hemostasis and cell culture, water purification, textile finishing, and composite materials (Ma et al., 2016; Wang et al., 2016). Keratin-based biomaterials have been created and used in many different disciplines, including films, hydrogels, dressings, and scaffolds (Luo et al., 2016).

In many locations throughout the New and Old World, porcupines have long been regarded as forest pests (Khan et al., 2000). The majority of porcupines are herbivores, and they favor some tree bark as well as bulbs, roots, and succulent tubers (Hafeez et al., 2011). Porcupine quills have antibiotic properties. When the quill’s structure is studied, keratin and fatty acids are revealed. The coating of the quills with free fatty acids is related to the antibiotic activity. It is believed that the fatty acids in porcupines’ quills help their antibacterial activity. Quills’ antibacterial characteristics might aid in preventing falls-related self-injury (Roze, Locke & Vatakis, 1990).

Majority of uses for keratin-based nanoparticles are in medication delivery. Nanotechnology based on keratin is developing daily, and its applications are growing daily as well. Such nanoparticles may one day be used in medicine as antibacterial theranostic agents (Fernando, Gunasekara & Holton, 2018). Due to the fact that many bacteria become resistant to various diseases, these particles are also being examined extensively as antimicrobial agents. These particles are being modified to improve the delivery of antibiotics in a secure manner. These are viewed as a substitute for reducing the bacterial multidrug resistance that has grown due to the overuse of antibiotics treatment globally.

The majority of applications for keratin-based particles are in the delivery of drugs. Nanotechnology is constantly evolving, and so are its potential uses. Since many bacteria develop resistance to different diseases, these particles are also being thoroughly studied as antimicrobial agents. To enhance the safe delivery of medicines, these particles are being changed. They are viewed as a replacement for cutting down on the global overuse of these therapies, which has led to an increase in bacterial multidrug resistance (Fernando, Gunasekara & Holton, 2018).

Researchers have applied keratin as a drug delivery system by utilizing the keratin nanoparticles loaded with medicines. The properties of the keratin nanoparticles like the high surface area, adjustable biodegradability, upgraded biocompatibility and an ease of functionalization have placed them among highly sought natural, cheap and recyclable biomaterials. The diversity of resources from animals are rich with keratin for example wool, human hair, and chicken feather *etc*. The keratin produced from these resources has been used to produce keratin nanoparticles through ionic gelation, self-assembly, nanoprecipitation, drug-induced aggregation, electrospray, desorption and other techniques (Ferroni & Varchi, 2021).

Due to its high biocompatibility, biodegradability, absorbability, and non-immunogenicity, keratin is a perfect material for a variety of drug administration methods. Because of the many disulfide, carboxyl, and amino group-containing polar side chains it has, keratin is very reactive chemically (Zhi et al., 2018). According to earlier research, doxorubicin-loaded keratin nanoparticles have been created (Aluigi et al., 2018). Docetaxel, an antimitotic medication, and chlorine 6, a photosensitizer, were produced using in-water synthesis (Gaio et al., 2019). Paclitaxel (Taxol), an anticancer medication, has also been encapsulated in keratin particles (Wimalasiri et al., 2021). Such research demonstrates how keratin particles can be loaded with labile medicines (Perotto et al., 2019).

Porcupine is considered a pest. The least is known about its economic importance in terms of the uses of its quills as an important biomass in the field of antimicrobials development. These quills are the waste when shed by porcupines routinely. The keratin extracted from these quills and then further turned into bioactive form is possible using the lipids extracted from the same quills. This provides an important step for the superior designing of a drug carrier and an adjuvant from natural keratin for antimicrobial drugs. This paper addressed these gaps and research work is focused on optimizing the extraction of the keratin from quills, and the formation of keratin microparticles. To further affect the bioactivity of the keratin microparticles, layered them with lipids extracted from the quills. Antioxidant, antibacterial, and drug adjuvant properties of these bioactive keratin microparticles were also finally investigated in this work.

## Materials and Methods

### Materials

Chloroform (95%), urea (granular), 2-Mercaptoethanol, 2, 2′-azino-bis(3-ethylbenzothiazoline-6-sulfonic acid) (ABTS), Muller Hinton Agar, were purchased from Sigma Aldrich (St. Louis, MO, USA). Methanol, polyvinyl Alcohol (PVA), acetone, sodium dodecyl sulphate (SDS), anhydrous sodium sulphate (), and dimethyl sulfoxide (DMSO) were purchased from Merck (Rahway, NJ, USA). All chemicals were supplied by local suppliers.  The erythromycin 250 mg (Indus Pharma, Pakistan) was purchased directly from a pharmacy shop in Muzaffarabad, Azad Kashmir.

### Collection of porcupine quills

Samples of both fresh and old porcupine quills were collected from different sites of porcupine habitat in the district Muzaffarabad (34.3551°N, 73.4769°E) of the State of Azad Jammu and Kashmir, Pakistan. Through a fortnightly field survey, we collected the quills that had been shed naturally, without human contact with the animal. The quills were kept in airtight containers labeled with information. These samples were kept in a refrigerator at a low temperature (4 °C).

### Extraction of keratin protein

The keratin extraction method with some modifications was used from earlier published research work (Yamauchi et al., 1996). The brief detail of the method with modifications is as follows. Samples of quills were cleaned overnight with agitation in a 1:1 mixture of methanol and acetone, then washed twice with Milli-Q water. Cleaned quills were allowed to air dry for the entire next day. Approximately, 25 g of the chopped quills were soaked in a solution containing 7 M of urea, 0.05 M of SDS, and 1.1 M of 2-mercaptoethanol to extract the keratin. The extraction of the keratin from quills was continued then at 60 °C for 5 h. The solution was then centrifuged at 10,000 rpm to extract the supernatant after being filtered through a 50-mesh stainless steel grid and redissolved in above solution for further 1 h. After addition of 2N HCl, the pH of hydrolysate solution was changed from 5 to 3, then a thick layer of precipitates started to settle at the bottom of the test tube (Gigliotti, Jaczynski & Tou, 2008). The precipitated keratin was washed with water and centrifuged three times for ten minutes at 12,000 rpm to eliminate salts and other contaminants. The precipitates of the keratin were then air dried and labeled as refined keratin. The yield of the keratin was measured according to following Eq. (1);

(1) \begin{eqnarray*}\text{Yield of Keratin Protein} \left( \text{%} \right) = \frac{\text{Amount of Extracted Keratin Protein} \left( \mathrm{g} \right) }{\text{Initial Amount of Porcupine' s Quills (g)}} \times 100\end{eqnarray*}

### Extraction of lipid

A modified version of the process described by Bligh & Dyer (1959) was used to extract the lipids from small pieces of porcupine quills. Distilled water, methanol, and chloroform were added in the ratios of 1:2:0.8 v/v. In order to separate the two phases, chloroform and water were added in a proportion of one-third of the total volume. A separating funnel was used to separate the lower chloroform phase, which was then collected in a screw-capped septum vial with anhydrous sodium sulphate and kept at 4 °C for later analysis. The amount of lipid was measured in mg/g of dry weight of porcupine’s quills. The average amount of lipids extracted was 4.12 mg/g.

### Synthesis of bioactive keratin microparticles

**Figure 1:**
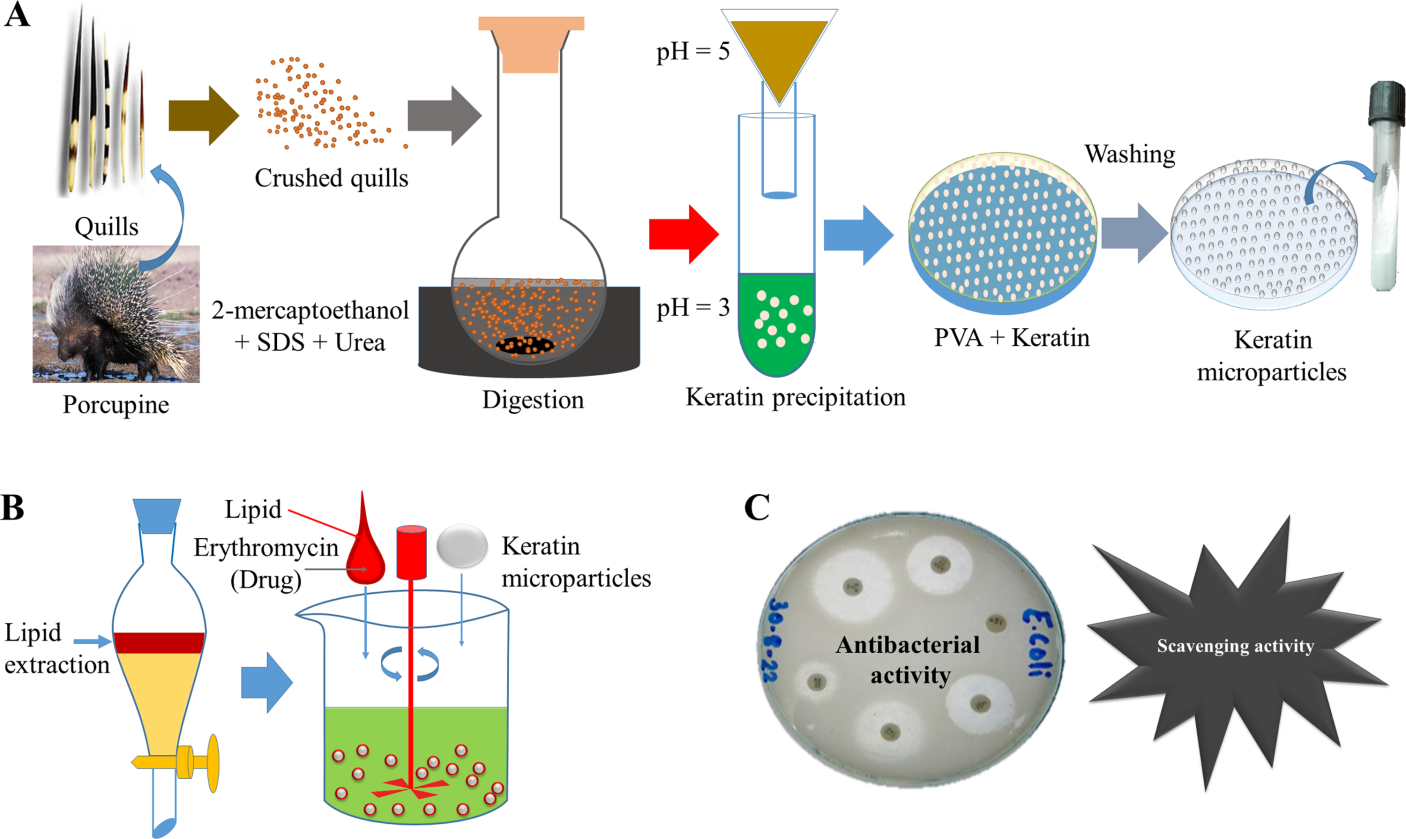
Scheme of study. (A) Extraction of keratin protein and synthesis of keratin microparticles with PVA, (B) Extraction of lipid and bioactivation of keratin microparticles with drug, (C) Antibacterial and scavenging (antioxidant) activities of bioactive keratin microparticles.

In Fig. 1, the detailed scheme is elaborated which was applied to prepare bioactive keratin microparticles. Keratin protein 50 mg mL^−1^ were added into PVA solution (3:1 w/w) and mixed for a minimum of 4 h at room temperature before casting into a petri dish and allowed to dry at room temperature. The resultant film was then washed with water to solubilize the PVA and to achieve the formation of keratin microparticles. These keratin microparticles were re-dispersed three times in Milli-Q water and centrifuged in order to obtain the pellet.

These keratin microparticles were turned bioactive by adding 3 w/v% of lipid extracted from porcupine quills. The lipid was added to keratin microparticles under continuous stirring at a low temperature, which was further followed by air drying. The 5% solution of drug erythromycin (antibiotic) treated with bioactive keratin particles for 18 h for drug loading. The following abbreviations were used for representing different compositions.

L—Lipid

E—Erythromycin

K—Keratin microparticles

LK—Lipid coated keratin microparticles

LKE—Lipid coated keratin microparticles (bioactive) loaded with erythromycin

### Response surface optimization of yield and size of the keratin microparticles

Box-Behnken design was adopted for response surface analysis of the independent variables *i.e.,* 2-mercaptoethanol, urea, reaction time and temperature for optimizing yield of the keratin and particle size of the keratin microparticles. Four independent variables were tested at three levels of inputs for each of 2-mercaptoethanol (1.5, 2.0, 2.5%), urea (6, 7.5 and 9%), reaction time (12, 18, 24 h) and temperature (90, 100, 110 °C) to map the response of dependent variables *i.e.,* yield (%) and particle size (µm) of the keratin. The Design-Expert 6.0.8 portable version (Stat-Ease, Inc. Minneapolis, MN, USA) was used to process the data of the dependent variables by fitting into the quadratic polynomial model. The analysis of variance (ANOVA) was selected to find out the significant difference in the interaction among the independent variables which significantly affected the response of the dependent variables.

### Surface analysis

Images captured under the optical microscope (Leica S6D Stereo Zoom Microscope, Wetzlar, Germany) were used to investigate the surface of L, K, LK, and LKE compositions. After applying 1 µl of each sample on the glass slide, samples were dried under laminar flow at room temperature. ImageJ software is a Java-based image processing application accessible from the National Institutes of Health webpage (https://imagej.nih.gov/ij/) was used for processing of the microscopy images. The size of the keratin microparticles was calculated after calibration of the scale bar on the captured microscopy images, and a bar chart with a distribution curve was used to display the variation in the size and distribution of the keratin microparticles.

### Chemical composition

Infrared spectra were measured after 50 µl of each sample mixed with KBr powder. Mixture was mixed well with pestle and mortar and pressed under pressure to obtain compact disc. The spectra of each composition was recorded using fourier transform infrared (FTIR) spectrometer (Shimadzu-8400S, Kyoto, Japan). All spectra were recorded in the range from 4,000 to 400 cm^−1^ with a resolution of 4 cm^−1^, and 32 scans.

### Scavenging activity

Scavenging activity of the keratin microparticles was assessed using ABTS (Mistry, Keum & Kim, 2016). Approximately, 180 mL of ABTS solution was mixed up with 20 mL of keratin microparticles’ solution with different doses of 0.1, 1, 10, 100, and 1,000 mg mL^−1^ and then incubated for 10 min in the dark environment. The absorbance at 734 nm was then determined through UV-Visible spectroscopy (T60UV-Visible Spectrophotometer, PG Instruments, Wibtoft, UK). Ascorbic acid served as the reference standard to evaluate the antioxidant activity of the keratin microparticles. The scavenging activity (%) was calculated from following Eq. (2)

(2) \begin{eqnarray*}\text{Scavenging activity}(\text{%})= \frac{\text{Absorbance of standard}\times \text{Absorbance of sample}}{\text{Absorbance of standard}} \times 100\end{eqnarray*}

The scavenging activity of the keratin microparticles was compared with scavenging activity of the ascorbic acid and the minimum inhibitory concentration (IC_50_) was calculated using following standard Eq. (3).

(3) \begin{eqnarray*}{\text{IC}}_{50}=(50-\mathrm{b})/\mathrm{a}.\end{eqnarray*}

Where b is the intercept and a is the slope of the given curve.

### *In vitro* antibacterial activity

LKE (load of erythromycin @ 5%) along with other compositions *i.e.,* K, L, and LK were tested through well diffusion method for *in vitro* antibacterial potency against the bacterial species—*Staphylococcus aureus* (gram-positive) and *Escherichia coli* (gram-negative). Antibacterial activity tested in triplicates for each composition.

Different concentrations of each composition were set to 20, 40 and 100 mg L^−1^ dissolved in DMSO and were loaded into wells formed on the Mueller Hinton Agar petri plates. Then, petri plates were incubated at 37 °C for 24 h. The zone of inhibition (ZOI) was measured by using vernier caliper (Mitutoyo, Japan). DMSO was used as a control in reference wells.

## Results and Discussion

### Keratin extraction and microparticles synthesis

**Figure 2:**
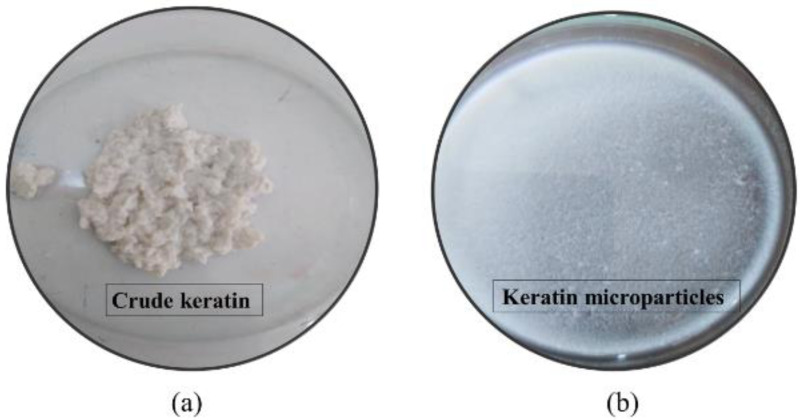
(A) Extracted crude keratin from *H. indica* quills at treatment condition set to mercaptoethanol 2%, Urea 7%, and reaction time 18 h; (B) Preparation of keratin microparticles from crude keratin after dispersion of keratin solution in 8% of PVA.

The protein keratin, which was taken out of porcupine quills, is depicted in its raw state in Fig. 2A. This keratin protein is a large, bulky protein mass that is whitish in color. Maximum keratin yield from quill waste was achieved at 120 °C, 7% urea, 18 h reaction duration, and 2% mercaptoethanol. As a result, the procedure and testing conditions were appropriate for obtaining keratin protein from porcupine quills.

The keratin microparticles, which were made from keratin protein that had been crudely purified, are shown in Fig. 2B. After the addition of 50 mg mL^−1^ of keratin protein into the PVA solution (3:1 w/w), and stirring for 4 h, the keratin was successfully dispersed. The isolated keratin protein was successfully fine-tuned into well-dispersed microparticles after the keratin dispersion in PVA solution. Microparticles with a minimum size of 69 µm were produced at conditions of 120 °C, 7% urea, 18 h reaction time, and 2% mercaptoethanol, whereas microparticles with a maximum size of 115 µm were seen at conditions of 18 h, 110 °C, 2% mercaptoethanol, and 7% urea. The extraction of keratin as a biopolymer from quill waste was therefore successfully proven in this work.

### Effect of extraction conditions

#### Temperature

**Figure 3:**
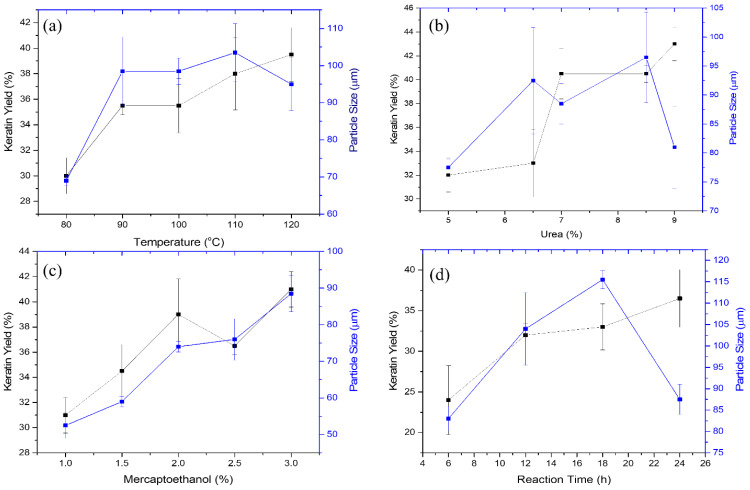
Effect of extraction conditions on the keratin yield and microparticle size. (A) Effect of temperature at fixed concentration of urea, 7%; reaction time, 18 h; mercaptoethanol, 2%, (B) Effect of urea under constant conditions of temperature, 110 °C; reaction time, 8 h; mercaptoethanol, 2%, (C) Effect of mercaptoethanol at temperature, 110 °C; reaction time, 8 h; and urea, 7%, (D) Effect of reaction time at constant conditions of temperature, 110 °C; mercaptoethanol, 2%; and urea, 7%.

The effects of varying the temperature settings (90, 100, 110, and 120 °C) on the extraction of crude keratin protein from porcupine quills and its impact on the size of keratin microparticles are depicted in Fig. 3A. The yield of keratin protein was discovered to increase generally with an increase in the reaction vessel’s temperature. The outer shell of the quill effectively protects the keratin located deeper within the cortical part of the quill. As a result, maintaining of the higher temperature may increase the disruption process and more efficiently release the keratin protein. At 80 °C, the keratin yield was 30%; at 90–100 °C, it increased further up to 35%, which is a 5% improvement. The increase in the keratin yield of 39.5%, was noticeable upon the temperature increase of 110 °C. Overall 9.5% improvement in the yield of the keratin protein from the porcupine’s quills was observed after gradually increasing the temperature under extraction conditions. The factors like, i) the difference in the discharge of amorphous and crystalline forms of the keratin from the cortex of the porcupine quills; and ii) possibly poor heat transfer at temperatures above 120 °C would have possibly caused barrier towards achieving higher yield than the estimated in this study.

The keratin protein’s microparticle size positively covaries with the extraction of the keratin. Figure 3A shows the change in the size of the keratin microparticles in response to the temperature changes. The increase of temperature from 80 to 110 °C, the size of the keratin microparticle increases. At 110 °C, the largest size of keratin microparticle was measured to be 103.5 µm. Keratin extracted at different temperatures was dispersed in PVA solution and noticed the difference in the sizes of the keratin microparticles. This difference demonstrates temperature affectes the keratin protein’s structural integrity during the extraction process. Due to the difference in the structure of the keratin, size variation was noticed during the formation of the keratin microparticles. PVA functions as a dispersant that lowers the interfacial tension between water and insoluble polymers (*i.e.,* keratin). The size of the microparticles increases in case of the keratin extracted at the higher temperature. Increase in the yield of the keratin at higher temperature may have involved irregular and bigger size of the keratin, which were dispersed poorly in the PVA solution. Hence, consequently, large size of keratin microparticles are generated. At 120 °C, the decrease in the size of the keratin microparticle was noticed. In such cases, higher temperature (*i.e.,* 120 °C) may have dissociated the more labile amorphous portion of the keratin protein from its more structured crystalline portion, which reduced the size of the keratin microparticles.

#### Urea

The effect of urea concentration on keratin protein extraction and subsequent production of keratin microparticles is depicted in Fig. 3B. The yield of keratin protein has increased due to the increase in urea concentration over the range of 5, 6.5, 7, 8.5, and 9%. The yield of the keratin protein does not significantly alter when the concentration of urea was increased by 5 to 6.5%. The keratin yield was maintained 22 to 23% at this concentration. The extraction of the keratin protein was improved by a further increase in urea concentration from 6.5 to 8.5%. The keratin protein output increased from 23 to 40% at these concentrations of urea, and it increased even more to a maximum of 43.5% at 9% of urea. According to these findings, the involvement of urea in the extraction of keratin from porcupine quills was best observed when the concentration of urea was adjusted above 6.5%.

The size of the keratin microparticles was also measured at various urea concentrations (Fig. 3B). The improvement in the size of those keratin microparticles was noticeable which were prepared from the keratin protein extracted with 6.5 to 8.5% of urea. When the concentration of urea was increased to 8.5%, the size of the detected keratin microparticles increased from 77.5 to 100 µm. The size of keratin micro particle is negatively impacted by the keratin protein extracted at 9% urea, and this was evidenced from the keratin microparticles observed size of 81 µm. This study’s findings suggest that urea may denature proteins at higher concentrations by reducing their hydrophobicity and interacting directly with the amide units by creating hydrogen bonds.

#### Effect of mercaptoethanol

The effect of mercaptoethanol on the yield of the protein extracted from keratin was examined as shown in Fig. 3C. It is found that increasing the amount of mercaptoethanol from 1 to 3% showed continuous improvement in keratin extraction. However, there is a linear relationship between an increase in mercaptoethanol concentration and a gradual improvement in the yield of the keratin protein up to 2% of mercaptoethanol. The yield pattern of the keratin at 2.5 and 3% of mercaptoethanol was inconsistent. Thus, the estimated yield might not be accurate or economical. At 3% mercaptoethanol, the highest yield of the keratin protein was attained 41%.

The size of the keratin microparticles and the amount of keratin produced by various mercaptoethanol treatments are shown to be closely highlighted in Fig. 3C. This indicates that the protein structure was affected more uniformly by the mercaptoethanol. This reduction in keratin microparticle size is made possible by the mercaptoethanol reducing capabilities, which helped to better cleave the disulphide link within the keratin protein during extraction. When compared to other conditional parameters investigated in this work, the particle size of 89 µm was obtained under such circumstances with a smaller size variation. The size of keratin microparticles was increased from 55 to 90 µm in response to increase in mercaptoethanol concentration. Hence, mercaptoethanol influence the sizing of the keratin microparticle under this study.

#### Reaction time

The impact of reaction time on the extraction of the keratin protein from porcupine quills is depicted in Fig. 3D. The yield of the keratin protein enhanced when the reaction time was prolonged from 6 to 24 h. The measured keratin output varied depending on the time, being 24% at 6 h, 32% at 12 h, 33% at 18 h, and 36.5% at 24 h. The extraction of the keratin protein benefited from the reaction time’s gradual rise. The yield of the keratin protein was notably high at 6 h, however at other periods the yield of keratin was not doubled by doubling the time. Yet, it was discovered that passing time had a different effect on how well the keratin protein could be extracted from the porcupine quill’s cortex.

The findings of keratin microparticles that were made from extracted keratin protein at various intervals have been presented in Fig. 3D. The isolated keratin proteins produced keratin microparticles with sizes of 83, 104, 115, 87 µm at 6, 12, 18, and 24 h, respectively. The trend is closely monitored from 6 to 24 h, which shows that time plays a key role in determining the size of keratin microparticles. After being extracted for up to 18 h, the keratin microparticles grew larger. This suggested that at 6, 12, and 18 h, the keratin protein fraction had a greater quantity of the protein, which could be the reason for poor dispersion in PVA and resulted in bigger size of the keratin microparticles. The smaller size of keratin microparticles (87 µm) after extraction at 24 h suggested that extended extraction times might have further shear the protein and encouraged the oxidation of sulfhydryl groups to occur.

### Response surface analysis

#### Keratin yield

The lowest proportion (27.26%) of keratin protein was produced by the independent variables mercaptoethanol 1.5%, urea 7.5%, reaction time 18 h and temperature 100 °C. The reaction mixture of 2.5% mercaptoethanol, 7.5% urea, 18 h at 110 °C, produced the best yield of keratin (42.25%). The important elements that contributed to the increase in keratin yield were the change in temperature from 100 to 110 °C and the increase in mercaptoethanol concentration from 1.5 to 2.5%. Mercaptoethanol, 2.5%; urea, 7.5%; reaction time, 24 h; temperature, 100 °C; and the largest size of the keratin microparticles achieved was 118.87 µm. The smallest size of the keratin microparticles, 60.5 µm, was produced with 1.5% of mercaptoethanol , 6% of urea, and 18 h of reaction period and 100 °C of temperature. The size of the keratin microparticles was affected by the concentration of mercaptoethanol, urea, and reaction time. Less stringent conditions probably helped to produce the smallest keratin microparticles.

**Figure 4:**
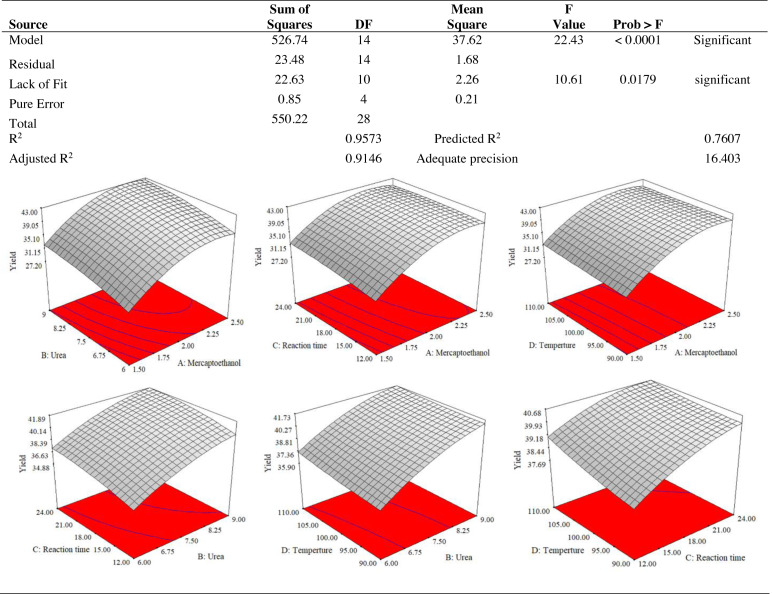
ANOVA for response surface quadratic model for keratin yield optimization.

The use of dependent and independent variables enabled statistical data processing to establish the response’s dependence on the independent variable. Based on the statistically analysed data for keratin yield and keratin microparticle size, Fig. 4 displays the analysis of variance (ANOVA). According to Table 1, the quadratic model of the keratin yield experiment had a *p*-value of 0.0001 (less than 0.05), indicating that it was significant. The model also had an F-value of 10.61, meaning that there was only a 1.79% possibility that a value this large could be the result of noise. This F-value highlighted the relevance of the model. The model had a robust enough signal to be used for optimization, as evidenced by the sufficient precision measure of 16.403 (more than 4) for the sample. Since they both had *p*-values of 0.0001, the mercaptoethanol and urea were significantly altering the keratin yield.

**Table 1:** Box-Behnken experimental design runs, factor levels and response variables.

**Run**	**Factors (X)**				**Response (Y)**	
	**X1**	**X2**	**X3**	**X4**	**Y1**	**Y2**
	**Mercaptoethanol (%)**	**Urea (%)**	**Reaction time (h)**	**Temperature (°C)**	**Keratin yield (%)**	**Particle size (µm)**
1	2	6	12	100	34.26	82.23
2	2	7.5	24	110	40.25	83.25
3	2	7.5	12	110	38.54	85.87
4	2.5	9	18	100	40.92	118.35
5	1.5	6	18	100	29.54	60.65
6	2	6	18	90	35.25	74.84
7	1.5	7.5	18	90	28.11	66.86
8	1.5	7.5	12	100	27.36	63.36
9	1.5	9	18	100	33.25	71.87
10	2	7.5	18	100	39.58	87.85
11	2	7.5	24	90	41.68	86.23
12	2	6	24	100	36.32	76.17
13	2	7.5	18	100	40.00	88.24
14	2	6	18	110	37.20	76.22
15	2	9	18	90	40.50	91.01
16	2.5	7.5	18	110	42.25	114.47
17	1.5	7.5	24	100	31.00	67.47
18	1.5	7.5	18	110	31.50	61.50
19	2.5	7.5	18	90	41.25	116.25
20	2	9	24	100	42.51	93.89
21	2	7.5	12	90	39.00	84.87
22	2	9	12	100	41.00	86.87
23	2.5	7.5	12	100	40.00	111.58
24	2.5	7.5	24	100	40.90	118.87
25	2	9	18	110	41.50	92.11
26	2	7.5	18	100	39.00	87.50
27	2.5	6	18	100	37.56	109.00
28	2	7.5	18	100	40.00	88.44
29	2	7.5	18	100	40.12	89.11

**Figure 5:**
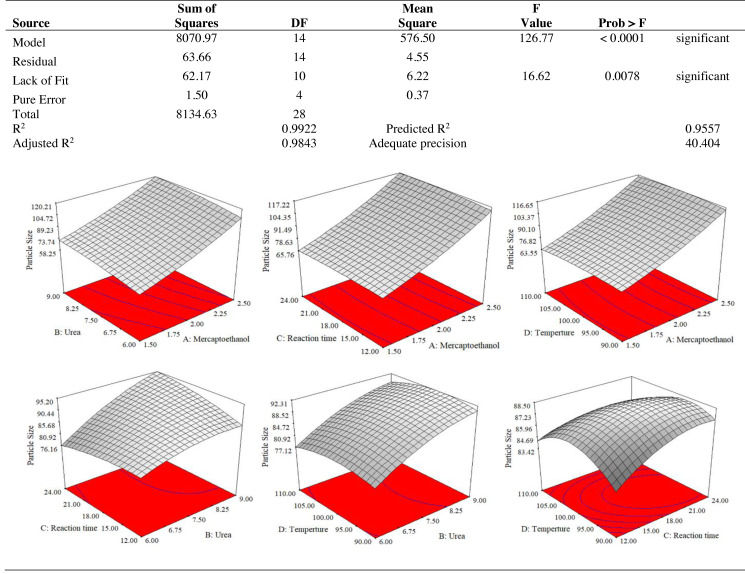
ANOVA for response surface quadratic model for keratin microparticle sizes optimization.

**Figure 6:**
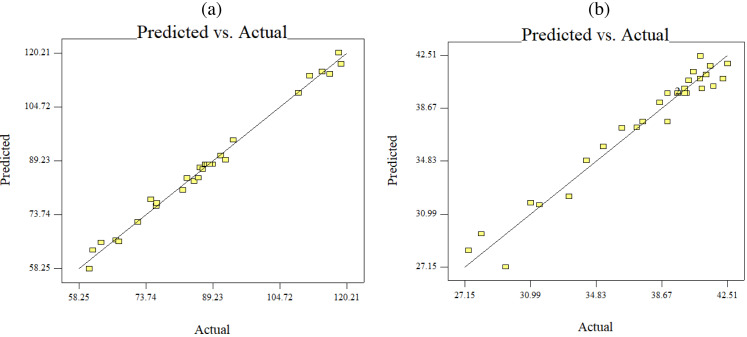
Predicted versus actual data fits using quadratic model, (B) keratin yield (B) keratin microparticles size.

The results of an ANOVA with interactions between several independent variables and the dependent variable, keratin microparticle sizes, are given in Fig. 5. A *p*-value of 0.0001 or less than 0.05 indicated the quadratic model’s relevance. Additionally, the model had an F-value of 16.62, which indicated that there was only a 0.78% possibility that a value this large could be the result of noise. This F-value highlighted the relevance of the model. Since they exhibited *p*-values of 0.0001, mercaptoethanol and urea were significantly impacting the keratin yield and keratin microparticle size response. Figure 6 shows that both experimental and predicted data for yield and size of the keratin microparticle fit well through applying the quadratic model through the response surface method.

### Surface morphology of keratin microparticles

**Figure 7:**
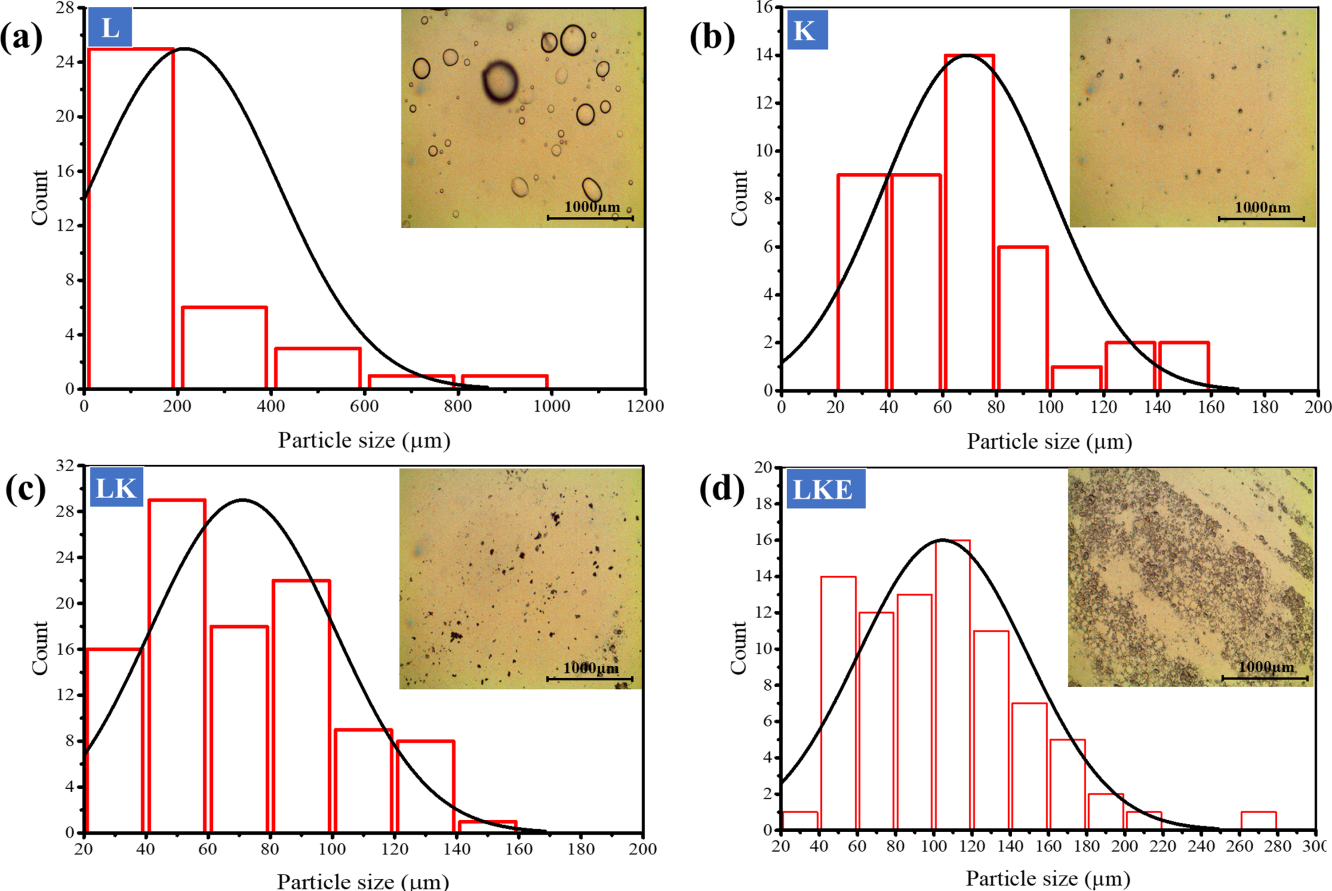
Optical microscopy study of shape, size and distribution of the keratin microparticles in aqueous solution at room temperature.

The shape, size and distribution of keratin microparticles were studied using optical microscopy (Fig. 7). Figure 7A, round shape of the lipid was used as reference of the shape assessment for keratin microparticles. In Fig. 7B, the shape assessment revealed that keratin microparticles were heterodisperse. This shape showed the difference from rounded to multifaceted morphology in the aqueous solution. The dried form of keratin microparticles showed the characteristics of specially dispersed and randomly loosely packed form. These observations are supported by earlier work (Rad, Tavanai & Moradi, 2012; Zhang et al., 2013). The size and distribution of the particles ranged up to 100 µm. However, the highest keratin microparticles count of 25% was noticed for the size of ≤200 µm.

### IR spectroscopy

**Figure 8:**
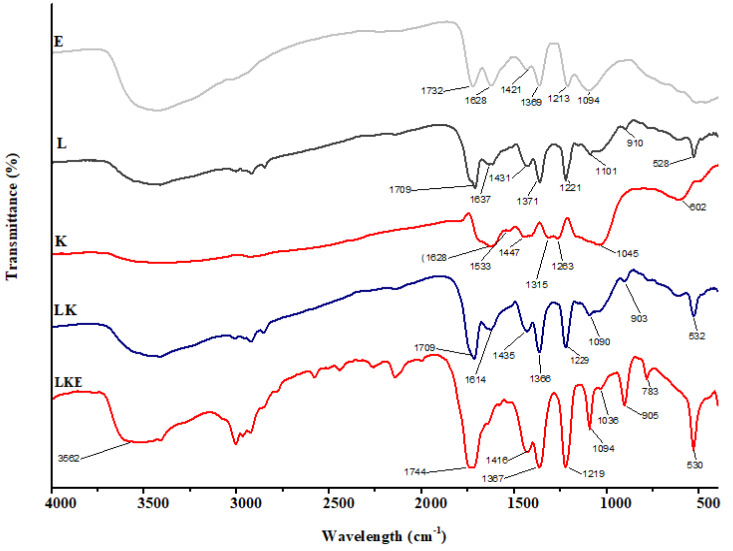
FTIR spectra of keratin microparticles.

The chemical makeup of the keratin microparticles and their various interactions with lipid and drug were determined using FTIR (Fig. 8). The results show that the chemical structure of keratin microparticles and their formulation with lipid and drug have changed. All of the examined materials’ absorbance spectra displayed the same pattern of keratin-specific peaks as in earlier studies (Ma et al., 2016).

The keratin samples displayed spectral bands that were designated as Amide A, Amide I–III and corresponded to peptide bonds (–CO–NH) (Xu et al., 2014; Sharma et al., 2017). Stretching vibration of –O–H and –N–H (Amide A) is responsible for the wide vibration band region between 3,400 cm^−1^ and 3,250 cm^−1^(Pavia et al., 2014). Stretching vibration of –NH bonds is linked to the Amide A band, which was observed at 3,280 cm^−1^. The stretching bonds in –CH are connected to the transmission bands that range from 3,000 to 2,700 cm^−1^. The –C =O stretching (Amide I) is responsible for the strong transmission band in the region of 1,628 cm^−1^ (Kakkar, Balaraman & Shanmugam, 2016). The C–H stretching and N–H bending are attributed to the Amide II vibration band in the range of 1,533 cm^−1^ (Eslahi, Dadashian & Nejad, 2013). Due to C–N stretching and N–H bending, the weak band between 1,315 cm^−1^ and 1,263 cm^−1^ corresponds to the Amide III band (Vasconcelos, Freddi & Cavaco-Paulo, 2008). The asymmetric and symmetric S–O stretching vibrations (cysteine-S-sulfonated residues), which are generated due to the interaction of sulphides and cysteine in the protein extraction process, respectively, are responsible for the absorption bands at 1,045 cm^−1^ (Aluigi et al., 2014). The successful coating of the keratin microparticles concealed the keratin signal in LK samples. Overlapping lipid and drug signals were seen in LKE. Thus, it was discovered that the surface of keratin microparticles had been successfully coated with drug-loaded in lipids.

### Antioxidant activities

**Figure 9:**
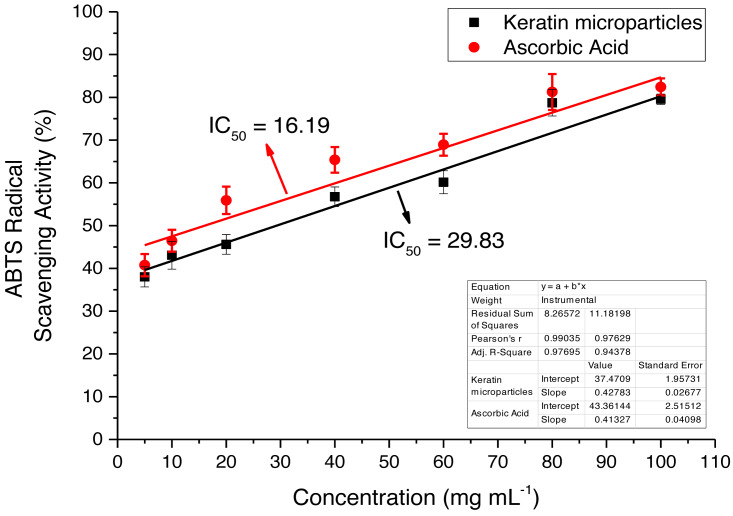
ABTS radical free scavenging activity for keratin microparticles and ascorbic acid (standard).

The extracted keratin microparticles were evaluated for the antioxidant activities by using ABTS radical free scavenging bioassay that showed the maximum absorbance at 734 nm The activity was investigated at different concentrations such as 10, 20, 40, 60, 80 and 100 mg mL^−1^ as shown in Fig. 9. Overall, highly significant antioxidant efficacies were observed for extracted keratin microparticles. The keratin microparticles had IC_50_ values of 29.83 mg mL^−1^ in ABTS bioassays, as compared to ascorbic acid value of 16.19 mg mL^−1^. The efficacies of keratin microparticles as antioxidant agents were higher as compared to ascorbic acid.

### Antibacterial activity and adjuvant effect of the keratin microparticles on drug efficacy

In Table 2, lipid extracted from the porcupine’s quills showed the ZOI values of 25.65 ± 0.54 mm against the *E. coli* which was higher than the ZOI noticed for *S. aureus* which was 24.54 ± 0.41 mm. Erythromycin showed almost the same ZOI values for *E.coli* and *S. aureus*. The keratin microparticles showed ZOI of 16.5 ± 0.71 mm for *E. coli* which was higher than the ZOI values of 15.75 for *S. aureus*. The keratin microparticles after lipid coating showed an activity of 23.50 ± 0.70 mm for *E. coli* and 25.75 ± 0.35 mm for *S. aureus.* The lipid coated keratin microparticles with loaded erythromycin have shown the activity of 43.00 ± 0.70 mm and 40.25 ± 0.35 mm for *E. coli* and *S. aurues* respectively. It is an interesting fact that the lipid coating of keratin microparticles and then loading of drug to lipid coated keratin microparticles increased the inhibitory effect due to the adjuvant effect of both the lipids and keratin microparticles on the drug erythromycin. Inhibitory activity of the keratin microparticles shifted approximatley 16.00 to 36.00 mm after lipid coating of the keratin microparticles. Therefore, a difference of 20 mm (55%) in the inhibitory activity which is a significant contribution. Similarly, the lipid coated keratin inhibitory activity was approximately 24 mm which increased to 42 mm after loading of drug erythromycin. This difference of inhibition was 18 mm (43%).

**Table 2:** The antibacterial activity of keratin microparticles with different compositions against clinical pathogenic bacteria *E. coli* and *S. aureus*.

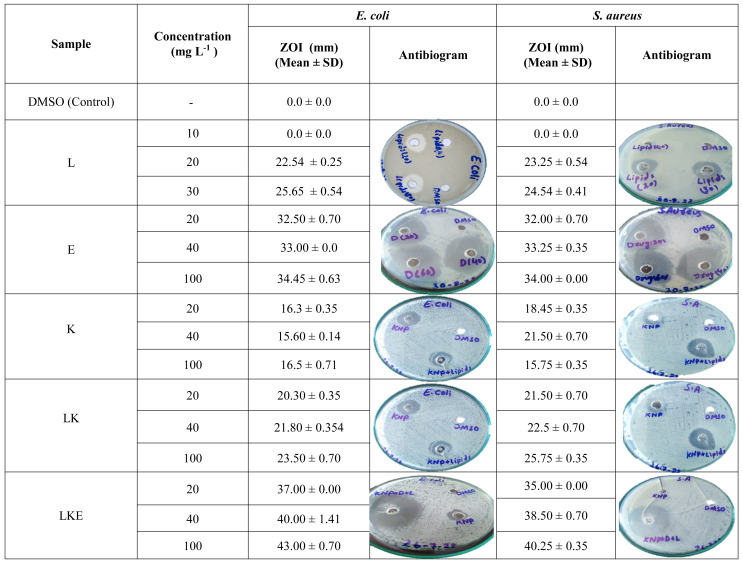

## Discussion

The properties of the keratin and its various formulations including nanoparticles have established *in vivo* and *in vitro* stability at physiological conditions, minimum immunogenicity responses and low synthesis cost (Pakdel et al., 2022). The interaction of the keratin that makes up porcupine quills gives them a great amount of hardness and strength. It is of significant interest to research on the extraction of keratin and the creation of microscopic microkeratin particles for usage as drug carriers or in adjuvant formulations for treating microbial resistance to antibiotics. With a maximum keratin yield of 41% and a minimum particle size of 27.1 µm, this work has successfully isolated the keratin protein (Figs. 2 and 3 and Table 1). Due to structural modifications involving the cylindrical shell, stiffeners, and foam, porcupine quills have considerable strength, making it difficult to extract the keratin (Ghazlan et al., 2021). A study using X-ray micro-computed tomography revealed that the stiffeners pointing in the direction of the core are connected radially to the thick outer shell (Tee et al., 2021). According to research on X-ray micro-diffraction of quills from African porcupines, the outer layer mostly contains moderately ordered *β*-keratin, the middle layer contains an amorphous and weakly ordered-keratin layer, and the inner layer contains highly ordered-keratin (Busson, Engström & Doucet, 1999). As a result, quill filaments are thought of as crystalline parts because they are made of firmly bound polypeptide chains (Bear & Rugo, 1951). As a result, urea and mercaptoethanol were used to remove the keratin from the quill. Mercaptoethanol served as a disulfide bond reducer and a sulfhydryl group protector, both of which helped extract the highest quality and most quantity of keratin from the intricate structure of quills (Ward & Lundgren, 1954). Urea is water-miscible and can be utilized as solvent to solubilize and recover proteins as documented for protein recovery from algae. It is well known that urea inhibits the precipitation and aggregation of proteins (Karan et al., 2019). It has been found that the rate of protein solubilization is directly proportional to the hydrolyzing agent concentration and temperature when keratin is extracted from sheep’s hair. The types and numbers of bonds in the polypeptide are primarily connected to the optimum parameters, which were discovered to be 80 °C, 0.5 N, and 3.5 h. The protein’s sulphide link is easily broken by temperature, allowing the protein to disintegrate (Mengistu, Angassa & Tessema, 2023). In other reports, the yield percentage and absorption of extracted keratin in hydrolysate increase in response to change in the extraction time, NaOH concentration, and pH (Teklemedhin, 2018). PVA’s reported function is that of a dispersant. PVA reduces the interfacial tension between polymers that are insoluble in water. The PVA’s emulsifying function improves the dispersion of keratin in film (Blehn & Ernsberger, 1948). PVA aids in the preparation and fine tuning of the keratin microparticles. Urea has the ability to dissolve both hydrogen bonds and hydrophobic interactions between and within proteins. When it is used in high concentrations, they dissolve proteins that would normally be insoluble, destroying the secondary protein structure (Chi & Cho, 2020). Mercaptoethanol, a potent reducing agent, effectively breaks and lowers disulfide bonds. It frequently breaks down tertiary and quaternary protein structures in addition to unfolding native proteins (Chen et al., 2022). As a result, urea and mercaptoethanol were discovered to be more significant when reaction time and temperature were evaluated by response surface optimization method. When disulphide bonds are broken under specific high/low pH circumstances but sustained high temperature, low molecular weight keratin is known to be formed (Smith, Blanchard & Lankford Jr, 1994). As a result, the microparticles in our work shrank from 105 to 95 µm in size due to the high temperature of 120 °C. At set temperature conditions, disulphide links in proteins result in little breaking of polypeptide bonds and maintain the molecular structure of keratin. Therefore, rise in the yield of higher molecular weight keratin is expectedly produced over the stepwise increase in the temperature (Eslahi, Dadashian & Nejad, 2013) which supported our results.

The previous studies keratin both high and low yields are cited for a chicken-derived keratin. The chicken derived keratin yield up to 82% has been reported (Sinkiewicz et al., 2017) while under various other chemical circumstances, the yields of keratin 18.3 to 33% have been assessed (Tonin et al., 2007; Kamarudin et al., 2017). The keratin components in chicken feathers, has revealed the cysteine-amino acid makes up about 3% of the protein (Chen & Li, 2012). Cysteine is the source of the 1.35% of sulphur which has been found in porcupine quills (Bekker & King, 1931) that can act as an antioxidant once it is liberated from the S-S bond. On the other hand, sulphenic acids (-SOH), which might be regarded as potent antioxidants, may be produced when alkaline reduction of chicken feathers takes place (Alahyaribeik & Ullah, 2020). In comparison to ascorbic acid, which had a value of 0.038 mg mL^−1^ in the DPPH (2,2-diphenyl-1-picryl-hydrazyl-hydrate) free radical experiment, keratin particles had an IC_50_ values ranging from 2.23 to 8.21 mg mL^−1^ for their ability to scavenge free radicals. Keratin had an IC_50_ that was higher than ascorbic acid. The potential of keratin microparticles to stop and restrict oxidative chain reactions, which may serve a biological purpose, has been demonstrated in this work.

With and without lipid or drug, there were obvious differences in the structure of the keratin microparticles. Microscopy research has however revealed an affinity between the keratin surface and the lipid (Fig. 7).

Drug penetrated into the lipid which coated on the surface of the keratin microparticles marked by the changes in morphology of the keratin microparticles. Hence, the surfaces of such keratin microparticles can change the loaded drug adjuvant properties. The keratin protein reactivity comes from its functional groups present on the peptide structures and residual amino acids. Particularly, the functional groups like NH_2_ (first-order amine group), COOH (carboxylic group) and S–S (disulfide bridges) all together makes the surface of the keratin based nanoparticles reactive (Pakdel et al., 2022). Free (unesterified) fatty acids, which make of 5.5 to 18.6% of the total lipids, are found in the quills’ covering. These free fatty acid fractions are linked to an inhibitory effect on gram positive bacteria as compared to gram negative bacteria (Roze, Locke & Vatakis, 1990). Therefore, our work also revealed the same findings as noticed by Roze, Locke & Vatakis (1990), that the antibiotic activity against the gram-positive bacterium *S. aureus* was comparable with the control and other formulations tested as shown in the Table 2. The current work has also looked into how to combat the gram-negative bacterium *E. coli*. The keratin microparticles activity was increased after the lipids were added to the surface of these microparticles. In literature, keratin coated with a lipid that contained a licochalcone A has shown better activity. To enhance the drug retention, a licochalcone A has been loaded into keratin liposomes with the aid of keratin lipids. Keratin liposomes effectively and without harm transferred licochalcone A into the cytoplasm of B16F10 cells (Wu et al., 2022). Free fatty acids are more polar because of their free –COOH end. These fatty acids interact nonpolarly with keratin (Brunner, 1962). In the most current research, non-polar contact was used to load the fatty acids onto the keratin surface. The keratinocytes’ capacity to successfully battle the tested bacterial strain is improved by these attachments. In our work, the erythromycin drug activity has been improved after interaction with the lipid activated keratin microparticles. This is supported with observed lipid coated keratin inhibitory activity of approximately 24 mm. This has been increased to 42 mm (43% improvement) after loading the drug erythromycin in the LKE composition. Thus the role of the lipid with the keratin improved the keratin microparticles properties, and moreover, adjuvant role of the LK for erythromycin has been witnessed in this research work. Thus, this research work recommends the emerging antibiotics resistance can be addressed by testing and applying keratin based designs to increase the potency and drug delivery to the targeted cells.

## Conclusion

It can be inferred from this work that keratin microparticles can be generated, bioactivated and employed as an adjuvant support for drug. The keratin microparticles with lipid coatings were successfully activated using lipids extracted from porcupine’s quills. The results of FTIR spectroscopy and microscopy were used to determine these compositions. These keratin microparticles had antioxidant and antibacterial characteristics. The suppression of the bacterial activity of *S. aureus* and *E. coli*, after treatment with keratin microparticles as well as with bioactivated keratin microparticles with lipid and drug have proved the keratin’s adjuvant characteristics for the drug efficacy which has promising pharmacological applications. The drug efficacy known as bioactive after loading into lipid-coated keratin served as the best pharmacological adjuvant. The mechanism of drug release and the stability from such keratin microparticles under various physiological *in vivo* settings should also be the subject of further research.

##  Supplemental Information

10.7717/peerj.15653/supp-1 Supplemental Information 1 Scavenging activity

10.7717/peerj.15653/supp-2 Supplemental Information 2 Yield and Particle Data

## Additional Information and Declarations

### Data Availability

The following information was supplied regarding data availability: The raw data are available in the Supplemental Files.
